# Prevalence of RhD status and clinical application of non-invasive prenatal determination of fetal *RHD* in maternal plasma: a 5 year experience in Cyprus

**DOI:** 10.1186/s13104-016-2002-x

**Published:** 2016-04-01

**Authors:** Thessalia Papasavva, Pete Martin, Tobias J. Legler, Marios Liasides, George Anastasiou, Agathoklis Christofides, Tasos Christodoulou, Sotos Demetriou, Prokopis Kerimis, Charis Kontos, George Leontiades, Demetris Papapetrou, Telis Patroclos, Marios Phylaktou, Nikos Zottis, Eleni Karitzie, Eleni Pavlou, Petros Kountouris, Barbera Veldhuisen, Ellen van der Schoot, Marina Kleanthous

**Affiliations:** Molecular Genetics Thalassaemia Department, The Cyprus Institute of Neurology and Genetics, 6 Internanional Airport Ave, Agios Dometios, 1683 Nicosia, Cyprus; International Blood Group Reference Laboratory, Bristol Institute for Transfusion Sciences, NHS Blood and Transport, North Bristol Park, Filton, Bristol, BS34 7QG UK; Department of Transfusion Medicine, University Medical Center Göttingen, Robert-Koch-Straße 40, 37075 Göttingen, Germany; Zoodochou Pigis Clinic, 9 Antisthenous, Kapsalos, 3086 Limassol, Cyprus; Mother and Child Medical Center, 9-11 Penelopis Delta Str., 1076 Nicosia, Cyprus; Ultrasound Department, Makarios Hospital, 6 Koritsas Str., Strovolos, Nicosia, Cyprus; Apollonion Private Hospital, Lefkotheou 20, Strovolos, 2054 Nicosia, Cyprus; European Woman’s Clinic, Vyzantiou 26, Strovolos, 2064 Nicosia, Cyprus; Ygia Polyclinic Private Hospital, 21, Nafpliou str., 3305 Limassol, Cyprus; Iasis Hospital, 8 Voriou Ipirou str., 8069 Paphos, Cyprus; G. Leontiades Clinic, 3 Apollonos Street, 6016 Larnaka, Cyprus; Patroklou Clinic, 14 Rubens Str., 3075 Limassol, Cyprus; Ledra Obstetrics Gynecology Clinic, 19 Pindarou, Ayios Antonios, 1060 Nicosia, Cyprus; Sanquin Blood Supply, PO Box 9892, 1006 AN Amsterdam, The Netherlands

**Keywords:** NIPD, Fetal *RHD* genotyping, RhD frequency, Cell-free fetal DNA

## Abstract

**Background:**

After the discovery that cell-free fetal DNA (cffDNA) is circulating in the maternal plasma of pregnant women, non-invasive prenatal diagnosis for fetal RhD in maternal plasma in RhD negative women at risk for haemolytic disease of the newborn (HDN) was clinically established and used by many laboratories. The objectives of this study are: (a) to assess the feasibility and report our experiences of the routine implementation of fetal *RHD* genotyping by analysis of cffDNA extracted from maternal plasma of RhD negative women at risk of HDN, and (b) to estimate the RhD phenotype frequencies, the *RHD* genotype frequencies and the RhD zygosity in the Cypriot population.

**Methods:**

cffDNA was extracted from maternal plasma of 73 RhD negative pregnant women. Real-Time Multiplex-PCR was used to amplify regions of *RHD* gene in exons 4, 5 and 10. RhD phenotypes were determined on 445 random samples using conventional agglutination slide test.

**Results:**

The fetus was predicted to be positive in 53 cases and negative in 18 cases. Two of cases were identified as D-variants, weak D type-1 and 11. The frequency of RhD negative homozygosity in the Cypriot population was estimated to be 7.2 %, while the frequencies of *RHD* hemizygosity and RhD positive homozygosity was calculated to be 39.2 and 53.6 %, respectively.

**Conclusion:**

Fetal *RHD* genotyping can be accurately determined using cffDNA from maternal plasma. The implementation of the test has eliminated all use of unnecessary anti-D and reduced the total use of anti-D by 25.3 % while achieving appropriate management of the RhD negative pregnancies.

## Background

The discovery that during pregnancy fetal DNA is circulating in maternal plasma and constitutes about 10 % of the total plasma DNA has opened up new avenues in non-invasive prenatal diagnosis (NIPD) [1–3]. This discovery led to the development on NIPD for fetal sex and fetal RhD during pregnancy [4–7]. The development of a reliable and sensitive assay is based on its ability to discriminate fetal DNA from the coexisting background of maternal DNA by detecting differences between the two. NIPD of fetal sex for X-linked disorders by performing real-time PCR using Y chromosome specific targets was one of the first clinical applications of NIPD [4, 8]. Soon after, NIPD for fetal RhD in maternal plasma in RhD negative women for the identification of pregnancies at risk for haemolytic disease of the newborn was clinically established and used by many laboratories [9–12].

**Fig. 1:**
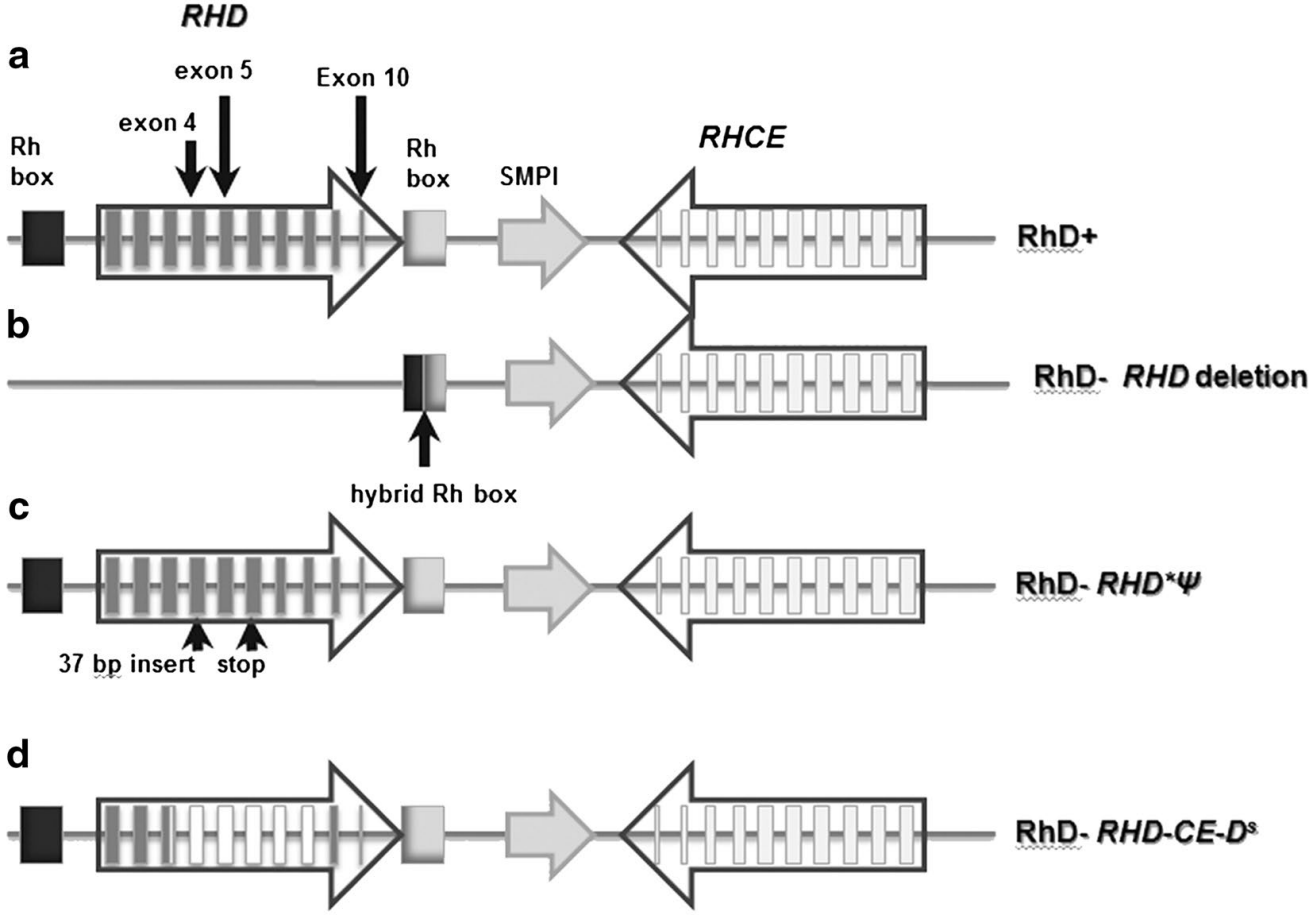
Schematic structure of the *RHD* locus. **a** Normal RhD positive individuals. **b** RhD negative individuals. **c** *RHD* pseudogene. **d** Hybrid *RHD*-*RHCE*-*RHD* ^*s*^ gene

*RHD* and *RHCE* genes located at chromosomal position 1p34.1-1p36 encode for the antigens of the Rh blood group. The genes are highly homologous encompassing 10 exons, while the *RHD* gene is flanked by two homologous DNA segments of the upstream and downstream *Rhesus boxes* [13, 14] (Fig. 1). In Caucasians, the RhD negative phenotype is usually caused by a deletion of the *RHD* gene that occurs by an unequal crossing-over between the Rhesus boxes leaving only a single hybrid *Rhesus box* [14].

The RhD negative phenotype is most common in Caucasians with a frequency of approximately 15 %, less common in Africans with 8 % frequency and rare in Asians with less than 1 % frequency [15]. About 66 % of RhD negative Africans have an inactive *RHD* gene, a pseudogene (RHD*Ψ) that results from a 37-bp insertion in exon 4 that introduces a stop codon, whereas about 16 % have the *RHD*-*CE*-*D*^*s*^ hybrid gene [16, 17]. About 0.2–1 % of Caucasians have red blood cells with a reduced expression of the D antigen (weak D) [18]. Single point mutations in *RHD* which encoding amino acid changes leading in a reduced number of D antigen sites on the red blood cells, are the main cause of weak D expression [19]. The identification of weak D phenotypes and genotyping is of clinical importance in terms of transfusion.

RhD negative pregnant women with a hemizygous RhD positive partner, have a 50 % chance of having a RhD negative fetus. If the fetus is RhD negative, there is no need for immunisation. However, if the fetus is RhD positive, the RhD negative woman may produce antibodies (alloimmunisation) to the fetal RhD antigens by silent fetomaternal haemorrhage during pregnancy, mostly during the third trimester and delivery, leading to the life threatening haemolytic disease of the fetus and newborn (HDFN) in a following pregnancy [20]. The injection of anti-D antibodies called Rh-prophylaxis to all RhD negative pregnant women does prevent this disease in most cases. However, many RhD negative women carry a RhD negative fetus and, thus, receive anti-D unnecessarily, exposing them to the risks associated with administration of human blood products that have been associated with serious viral infections in the past [21].

The clinical application of the non-invasive test for fetal RhD typing was implemented soon after the discovery of circulating fetal DNA. NIPD of fetal *RHD* from maternal plasma of RhD negative mothers is considered a valuable tool in the identification of pregnancies at risk of HDFN and the proper management of these pregnancies, but mainly avoiding unnecessary routine RhD prophylaxis [22–24]. Since 2001, NIPD of fetal *RHD* has already been offered to pregnant women with RhD positive partners as a routine clinical service by the International Blood Group Reference Laboratory [25] with other European countries following [10, 26–29].

This report discusses our experiences of the routine implementation in clinical practice of fetal *RhD* genotyping from the maternal plasma of RhD negative mothers in Cyprus. NIPD of fetal *RHD* was performed on maternal plasma on 71 RhD negative women, referred by their obstetrician during their routine visit, targeting exons 4, 5 and 10 of the *RHD* gene using Real Time PCR. The child’s RhD phenotype was confirmed serologically after birth. There is an indication of a relatively high percentage of RhD negative people in the Cypriot population based on the empirical observation of the gynecologists. In this work, the RhD phenotype frequency was examined and, for the first time, the RhD negative frequency was estimated for the Cypriot population, giving an assessment of how many couples are at risk for HDN and in turn eligible for NIPD every year.

## Methods

### Sample collection

Fifty maternal plasma samples from RhD negative pregnant women previously typed were sent by the University Medical Center Göttingen in Germany to be used for the verification of the NIPD assay already validated by the International Blood Group Reference Laboratory. Seventy-three RhD negative pregnant women with RhD positive partners at risk of HDN were referred by their obstetrician, during their routine visit, to the Cyprus Institute of Neurology and Genetics for the NIPD of fetal RHD using maternal plasma. These women were from the entire Cyprus. Nine milliliters of peripheral blood was collected in EDTA tubes after the 16th week of gestation.

Peripheral blood from 445 unrelated adults of both sexes, 222 males and 223 females, were drawn into EDTA tubes for the determination of RhD phenotype. All participants were Greek Cypriots, β-thalassaemia carriers referred to the Cyprus Institute of Neurology and Genetics between the period 2009–2010 for β-thalassaemia prenatal diagnosis purposes from the entire Cyprus. β-thalassaemia trait is not associated with RhD expression inheritance since the genes are located in different chromosomes.

Written informed consent was obtained from all maternal plasma samples from RhD negative pregnant women sent by the University Medical Center Göttingen in Germany for validation purposes. RhD negative pregnant women included in the study provided informed consent for the routine clinical analysis, Non-Invasive Prenatal Determination of Fetal *RHD* in maternal plasma. The test is approved by the Board of Directors of the CING and accredited by CYS EN ISO 15189:2007. All whole blood samples were collected as part of a routine patient care after the request of the participants. The study was performed as part of our diagnostic services.

### DNA isolation

For the isolation of cell-free maternal plasma from whole blood, the samples were subjected to centrifugation at low speed, 2500*g* for 10 min without brake. Subsequently, maternal plasma was transferred to microcentrifuge tubes and subjected to a second centrifugation step at 16,000*g* for 40 min to remove any residual cells. The maternal plasma was carefully removed and transferred into polypropylene cryogenic vials in 1 ml aliquots and stored at −20 °C until further processing.

Cell-free DNA was extracted from 1 ml of maternal plasma before DNA analysis using QIAamp Circulating Nucleic Acid Kit (Qiagen GmbH, Hilden, Germany) according to the manufacturer’s instructions.

Genomic DNA was isolated from 1 ml of peripheral blood using the Puregene Blood Core Kit C (Qiagen Sciences, Germantown, MD, USA).

### RhD phenotyping

RhD antigen testing was performed by conventional slide test using anti-D murine monoclonal blend, Molter-clone (Ortho Clinical Diagnostics, Inc., Raritan, NJ). The phenotyping test was performed on 445 randomly selected samples from the Greek Cypriot population. The genotype frequencies and zygosity were calculated, as described in the “Results” section.

### Fetal RhD Genotyping using real time polymerase chain reaction (PCR)

The NIPD assay for fetal RhD implemented at our lab has been designed, validated and applied by Finning et al. at the International Blood Group Reference Laboratory in Bristol [22, 25]. Verification of the assay was performed by using fifty maternal plasma samples from RhD negative pregnant woman, previously typed and sent by the University Medical Center Göttingen in Germany. Real Time multiplex PCR was used to amplify the regions of *RHD* gene in exons 4, 5, and 10, using 4 replicates per exon per sample (Fig. 1). The exon 4 and 5 assays are designed to amplify only *RHD* and not RHD*Ψ. Amplification of the *SRY* gene on the Y chromosome is used to confirm the presence of male fetal DNA. Amplification of the *CCR5* gene, common to mother and fetus, is used to evaluate the efficiency of the DNA extraction procedure and to estimate the total quantity of DNA (both maternal and fetal) present in maternal plasma. Four replicates for each exon are performed in order to improve sensitivity and to verify the accuracy of the fetal RhD status. During each run, genomic DNA from RhD negative female as well as RhD positive male were used for negative and positive control respectively. DNA sample from a person having the *RHD* pseudogene was also incorporated in the assay to exclude the possibility of someone carrying the pseudogene. Moreover, genomic DNA from the parents was also included for genotyping in order to confirm the phenotype.

### Multiplex ligation-dependent probe analysis (MLPA)

To identify *RHD*-variants in two pregnant women, the MLPA assay was performed as previously described [30]. The MLPA fragments were analysed using a 3130 Genetic Analyzer (Applied Biosystems) and Genemarker software version 1.85 (Softgenetics, State Collage, USA). A DNA sample with a RhD positive phenotype and an artificial control sample consisting of a mixture of a human cell line and plasmid DNA, containing all targets within the MLPA assay were used as controls.

### Statistical analysis

The data were analysed using the R programming language (version 3.1.2). Descriptive statistics were utilised for the analysis, while the confidence intervals (CI) were defined using the exact binomial test (function binom.test() in R). In cases where no negative effects were observed, such as the method’s accuracy, the 95 % CI was estimated using the rule of three [31].

## Results

### Determination of RHD allele frequencies in Cyprus

Random samples from 445 people from the Greek Cypriot population were tested serologically using standard haemagglutination assays to examine the phenotype and to determine the frequency of the RhD status in the Cypriot population. It was found that 32 individuals showed no agglutination with the RhD antigen, thus having negative RhD phenotype. The 32 RhD negative identified individuals were confirmed using PCR with restriction fragment length polymorphism (PCR–RFLP) as previously described [32]. The other 413 samples demonstrated positive agglutination and hence RhD positive (Table 1). Therefore, 7.2 % of the population in Cyprus is RhD negative (95 % CI 4.97–10.00 %). Using the Hardy–Weinberg equation, $p^{2} + 2pq + q^{2} = 1$ with *q*^*2*^, = 0.0719 (95 % CI 0.0497–0.1), the homozygous RHD, *p*^*2*^, is calculated to be 0.536 (95 % CI 0.468–0.604). Similarly, the hemizygous RhD frequency, *2pq*, is calculated to be 0.392 (95 % CI 0.347–0.433). These data were used to determine the *RHD* zygosity, *z*, which is defined as the fraction of hemizygous *RHD* samples over the homozygous *RHD* plus hemizygous *RHD* samples given by:

$$z = \frac{hemizygousRHD}{\hom ozygousRHDpositive + hemizygousRHD}$$

**Table 1:** RhD phenotype frequencies in 445 randomly selected samples from the Greek-Cypriot population

RhD determination	No.	Frequencies (%)	95 % CI
*RhD positive*	413	92.81	90.00–95.03
*RhD* negative	32	7.19	4.97–10.00
Total	445		

The frequency of individuals that are *RHD* hemizygous would be 42 % (95 % CI 36–48 %) among individuals that are RhD positive. Based on this result, a couple with the mother being homozygous *RHD* negative and the father hemizygous *RHD* positive has a 21 % (95 % CI 18–24 %) reproductive chance of having a child homozygous *RHD* negative and, therefore, not at risk of HDFN since the newborn will share the same genotype with the mother. As a result, these women take unnecessary immunoglobulin treatment.

### NIPD for Fetal RhD using Real Time Polymerase Chain Reaction

The RhD status of the fetus in 71 pregnancies was determined by real-time PCR using fetal DNA derived from maternal plasma. If no *RHD* signals are obtained for exons 4, 5 and 10, the fetus is determined to be RhD negative. The fetus is determined as RhD positive when at least two positive signals are obtained from each *RHD* exon plus a total of three more positive signals from any exon. No grey zone results were observed. The presence of the maternal DNA was observed in all cases. Based on the above criteria, the fetus was predicted to be positive in 53 cases and negative in 18 cases (Table 2). As a result, these women did not receive any anti-D prophylaxis and, therefore, a 25.3 % reduction in anti-D prophylaxis was achieved. Moreover, a better and closer monitoring was provided to the RhD positive pregnancies.

**Table 2:** NIPD of fetal *RHD* on maternal plasma of RhD negative pregnant women

Fetal RhD determination	No.(%) of patients	Serological confirmation on the newborn’s phenotype
Positive	53 (74.6)	53
Negative	18 (25.3)	18
Total	71	71

### Identification of D variant cases

During the routine fetal RhD genotyping two discrepancies were observed. In both cases, the mother, being serologically negative was referred to us for NIPD of fetal RhD from maternal plasma. However, upon genotyping the maternal genomic DNA sample with Real Time PCR, *RHD* sequences were detected in the mother’s sample indicating that the mother is RhD positive. In order to confirm this discrepancy, PCR-Restriction Enzyme Digestion was also performed on the maternal genomic DNA samples to test for RhD zygosity. Both cases were found to be hemizygous. Therefore, with the aim of investigating these serologic discrepancies in RhD typing, the samples were referred to Sanquin center in Amsterdam to investigate for possible RhD variants. Multiplex Ligation Probe Amplification (MLPA) was performed on both samples where it was found that sample 1 was weak D type 1/d Cc ee (RHD*01 *W.01*) having the nucleotide change 809T > G, while sample 2 was weak D type 11/d Cc ee (RHD*11) with nucleotide change 885G > T. These D variants have a reduced expression of the RhD antigen that arise from single point mutations on the *RHD* gene encoding amino acid changes and, hence, no agglutination is performed explaining the negative serology result. Therefore, NIPD cannot be performed on these samples since *RHD* sequences are present in the mother’s genome.

### Confirmation of fetal RhD status

Serological tests on the infant’s red blood cells (RBCs) were performed and RhD type was confirmed after delivery on all samples tested after contact with the referring physician and receipt of written confirmation of the newborn’s serological analysis (Table 2). When compared to postpartum serological results, an accuracy rate of 100 % (95 % CI 95.3–100 %) was achieved in our prenatal prediction of fetal RhD status. No false negative or false positive results were obtained on the 71 samples that were cross checked.

## Discussion

In this study, we have determined the RhD phenotype status of 445 random samples in an attempt to establish the frequency of the *RHD* negative allele in the Cypriot population. As a result, the frequency of the RhD negative phenotype in the Cypriot population is found to be 7.2 % (95 % CI 5–10 %), a value that is demonstrated to be different from that reported in the literature where it is stated that about 15 % of Caucasians do not express the RhD antigen. However, it is more consistent with 8.59 % frequency reported for the Greek population [33]. In non-European populations the frequency of the RhD negatives is much lower than Caucasians with Africans having 8 %, higher than the Cypriots, and Asians 1 % [15].It is reported that in western European populations the frequency is greater, the highest for instance of 21–36 % in the Basque French population [34]. The determination of the frequency of *RHD* negative alleles in Cyprus it is also important as it gives an indication, of how many couples are at risk for HDFN, a number that differs from what applies to Caucasians, and therefore in need for the prenatal determination of fetal *RHD* with maternal plasma. The determination of the paternal *RHD* zygosity is valuable in clinical setting as it can aid in the assessment of a couple’s risk of carrying a RhD positive child and in turn in risk of HDFN related to anti-D. The reported frequency of *RHD* hemizygosity would be around 56 % [35], for the Caucasians, however our study demonstrated a frequency zygosity value of 42 % among individuals that are RhD positive, in the Cypriot population, a value that is consistent with the lower RhD negative phenotype observed. Based on our results, fathers who are *RHD* positive have a 21 % predicted chance of a homozygous RhD negative fetus. Hemizygous *RHD* males have a 50 % chance of having a RhD negative offspring, in which case the pregnancy will not be at risk for HDFN.

Several studies have demonstrated the feasibility and accuracy of prenatal determination of fetal *RHD* with maternal plasma. A number of centers worldwide have reported the introduction of the test in their clinical practice. We have also applied the non-invasive fetal RhD genotyping in maternal plasma in a routine diagnostic setting in our lab and we have tested 71 samples since 2009. Our results were confirmed with the serological test on the newborns red cells on all samples tested. With the implementation of this method into routine clinical practice, we have achieved 25.3 % reduction in the administration of anti-D since 18 out of the 71 RhD negative women were predicted to have a RhD negative fetus. The observed difference, although relatively close, between the achieved value of 25.3 %, and the predicted one, 21 %, could be related to the small number of maternal plasma samples analysed compared to the 445 samples from which the predicted value was derived. Nevertheless, the implementation of the non-invasive fetal RhD testing and the high accuracy rate achieved is of paramount importance as it allows targeting immunoprophylaxis only to RhD negative pregnant women carrying an RhD positive fetus. Therefore, unnecessary administration of Ig anti-D to those carrying RhD negative fetus is avoided. Moreover, the implementation of this test not only serves as an important tool for antenatal diagnosis of fetomaternal incompatibility but also allows for a better follow-up and management of anti-D immunised women.

Based on our RhD phenotyping results, around 600 pregnancies per year in Cyprus are at risk for HDNF (RhD negative pregnant women with RhD positive partners), therefore eligible for NIPD for fetal RhD. It was observed, however, that the actual implementation of the programme into routine practice was slow with an initial number of samples far below the expected number. This might be attributed to the fact that this a fairly new test and currently the cost is not covered by the national health system. Moreover, clinicians sometimes are skeptical in embracing and adopting a new approach. We anticipate that with continuous education and a possible health care system that will cover the cost, an improvement in participation of more samples will be achieved.

Two out of the 73 samples referred for NIPD were identified as RhD variants, specifically weak D type 1 and 11. Weak D types result from nucleotide changes that encode amino acid substitutions in the membrane or below the membrane of the RhD polypeptide [36]. These changes affect the efficiency of insertion and, in turn, the quantity of RhD protein in the membrane reducing the number of RhD antigen sites on the red blood cells [19] leading to lack of reactivity with anti-D, no agglutination is performed supporting the RhD negative phenotype. Although the haemagglutination assay shows a sensitivity and specificity of 99.5–100 %, it is accepted in the literature that weak D units may escape detection by serologic methods. However, serology is still widely used to assign RhD negative or RhD positive patient [37–39].An estimated 0.2–1 % of Caucasians carry red cells with a reduced expression of the RhD antigen (weak D) [18, 39] as reported in the literature. Our experience shows that about 2.7 % (95 % CI 0.33–9.55 %) of RhD negative women carry a weak D type genotype, but a larger sample size is needed to determine whether this difference is significant. Weak D types 1–4 represent more than 90 % of all weak D occurring in Europeans [40]. Appropriate assignment of RhD antigen status is critical since commercially available anti-D reagents react differently with D variants. Patients with a weak D type 1 are treated as RhD positive, therefore transfused with RhD positive RBCs and as a result RhIg is not administered to pregnant women. On the other hand weak D type 11 should be treated as RhD negative, transfused with RhD negative RBCs and therefore RhIg is administered to pregnant women [19, 30, 40]. The above haemotherapy approach is in accordance with guidelines in Europe [40], however, in Cyprus there are no guidelines, since all weak D types are managed as RhD negative. The identification of weak D types and their classification will assist in the development of a more efficient guidance and management of an RhD negative transfusion policy in Cyprus. Therefore, a collaborative study with all the relevant health care officials is highly recommended.

## Conclusions

In this study, we have demonstrated the diagnostic accuracy of our NIPD test for identification of the fetal RhD status in maternal plasma and its importance in managing the RhD negative pregnancies. We have also demonstrated that the introduction of this test in clinical setting has eliminated the use of unnecessary anti-D and reduced the total use of anti-D by 25.3 %. Through the study, RhD variants were identified and defined. Moreover, we have estimated the RhD phenotype frequencies in the Cypriot population to be 7.2 % (95 % CI 5–10 %) for RhD negative and 92.8 % (95 % CI 90–95 %) for RhD positive. In addition, we have calculated the RHD genotypes in the Cypriot population to be 53.6 % (95 % CI 46.8–60.4 %) for RHD positive homozygous and 39.2 % (95 % CI 34.7–43.3 %) for RHD hemizygous. Finally, it is recommended that a systematic and targeted prevention based on fetal RhD genotyping from maternal plasma should be evaluated to define the impact and potential benefits on management, and quality of life.

## Abbreviations

cffDNA: cell-free fetal DNA
HDN: haemolytic disease of the newborn
NIPD: non invasive prenatal diagnosis
HDFN: haemolytic disease of the fetus and newborn
MLPA: multiplex ligation-dependent probe analysis
PCR: polymerase chain reaction
RBCs: red blood cells
